# Chinese Herbal Medicine and Its Regulatory Effects on Tumor Related T Cells

**DOI:** 10.3389/fphar.2020.00492

**Published:** 2020-04-21

**Authors:** Robert D. Hoffman, Chang-Yu Li, Kai He, Xiaoxing Wu, Bai-Cheng He, Tong-Chuan He, Jian-Li Gao

**Affiliations:** ^1^International Education College, Zhejiang Chinese Medical University, Hangzhou, China; ^2^DAOM Department, Five Branches University, San Jose, CA, United States; ^3^Academy of Traditional Chinese Medicine, Zhejiang Chinese Medical University, Hangzhou, China; ^4^The First Affiliated Hospital, Zhejiang University, Hangzhou, China; ^5^Molecular Oncology Laboratory, Department of Orthopaedic Surgery and Rehabilitation Medicine, The University of Chicago Medical Center, Chicago, IL, United States; ^6^School of Pharmacy, Chongqing Medical University, Chongqing, China

**Keywords:** T cells, tumor, traditional Chinese medicine, Chinese medicine, immunotherapy

## Abstract

Traditional Chinese medicine is an accepted and integral part of clinical cancer management alongside Western medicine in China. However, historically TCM physicians were unaware of the chemical constituents of their formulations, and the specific biological targets in the body. Through HPLC, flow cytometry, and other processes, researchers now have a much clearer picture of how herbal medicine works in conjunction with the immune system in cancer therapy. Among them, the regulation of tumor-related T cells plays the most important role in modulating tumor immunity by traditional Chinese medicine. Encouraging results have been well-documented, including an increase in T cell production along with their associated cytokines, enhanced regulation of Tregs and important T cell ratios, the formation and function of Tregs in tumor microenvironments, and the promotion of the number and function of normal T Cells to reduce conventional cancer therapy side effects. Chinese herbal medicine represents a rich field of research from which to draw further inspiration for future studies. While promising agents have already been identified, the vast majority of Chinese herbal mechanisms remain undiscovered. In this review, we summarize the effects and mechanisms of specific Chinese herbs and herbal decoctions on tumor related T cells.

## Introduction

Traditional Chinese medicine (TCM) has utilized herbs and herbal extracts to treat a variety of diseases and disorders for over 2000 years. Among these diseases, Chinese doctors have treated masses and tumors through the prescription of various herbs and formulations to reduce symptoms describing the etiology of cancer. As cancer is one of the leading causes of death in China (He J. et al., 2005), significant resources have been devoted to study the effects of TCM alongside Western medical treatments. Clinical and preclinical trials have demonstrated the efficacy of Chinese medicinal herbs as adjuvant treatments by enhancing the effects of conventional cancer therapies, reducing toxicity, and improving quality of life factors (Nie et al., 2016; Xiang et al., 2019).

Cancer was first described during the Shang dynasty (6^th^ –11^th^ century BC) where it was described as liú, tumor, or lump (Liu et al., 2015; Xiao, 2018). Clinical manifestations were described in medical literature as early as 400 BC with further elucidation by Wei Ji Bao Shu in 1170 AD, and Yang S.Y. in 1264 AD (Hu et al., 2012; Li et al., 2013). But, it is in the classic text, the Huang Di Nei Jing (HDNJ) or Yellow Emperor’s Inner Classic, circa 250 BC, that the full clinical picture of cancer is presented.

Today, TCM is an accepted and important part of cancer treatment alongside Western medicine in China and is gaining acceptance in the United States. (Liu et al., 2015; Ling et al., 2018; Wang Q. et al., 2017; Xiang et al., 2019). Complementary medicine has increased 30% in National Cancer Institute (NCI) designated cancer centers, and acupuncture is represented in 73% of those centers, herbal medicine in 66.7% (Yun et al., 2017). Chinese herbs are typically used as an adjuvant therapy, and research suggests the alleviation of clinical symptoms, such as pain, and prolonged survival time of post-operational, and advanced stage cancer patients (Zhang et al., 2017).

Within TCM, treatment methods are inextricably linked to pattern-differentiation (BIAN ZHENG). This differential diagnosis is based on four distinct examination methods—observation, palpation (Chinese pulse methods), interrogation, hearing, and smelling. These diagnoses are dependent upon the clinician’s subjective judgement and clinical experience, which can induce considerable variability in clinical trials and evidence-based research (Hsiao and Liu, 2010). Further, a western diagnosis of cancer may present as a variety of TCM patterns for which the clinician might devise a treatment plan (Ferreira and Lopes, 2011; Jiang et al., 2012). As such, TCM treatments are very specific and the emphasis is on individual therapy (Hsiao and Liu, 2010).

TCM practitioners may consult with a patient’s oncologist in devising a treatment plan; however, the TCM strategy may also be conceived completely outside the biomedical treatment program as TCM practitioners in the United States most often work in private practice, removed from institutional settings (NCCAOM.org). A typical TCM treatment would involve the aforementioned differential diagnosis, followed by an acupuncture protocol involving one or more needles designed to strengthen the patient’s immune system, alleviate current side effects resulting from chemo or radio therapy, and in some cases, reduce masses (Lu et al., 2008). The specific acu-points used would be determined by traditional functions and indications as indicated in classical texts such as the *Zhenjiu Da Cheng* and the *Huangdi Neijing Ling Shu*. The practitioner would then determine if an herbal formula is suited to the patient’s diagnosis. Once again, the formula chosen would be based on differential diagnosis and classical Chinese medicine texts such as the *Shang Han Lun* and *Jin Gui Yao Lue*. In some cases, TCM practitioners may have advanced training through a doctoral program or specialty centers, such as Memorial Sloan Kettering’s Cancer Center, from which they may have additional sources for choosing treatment protocols (mskcc.org).

**Figure 1:**
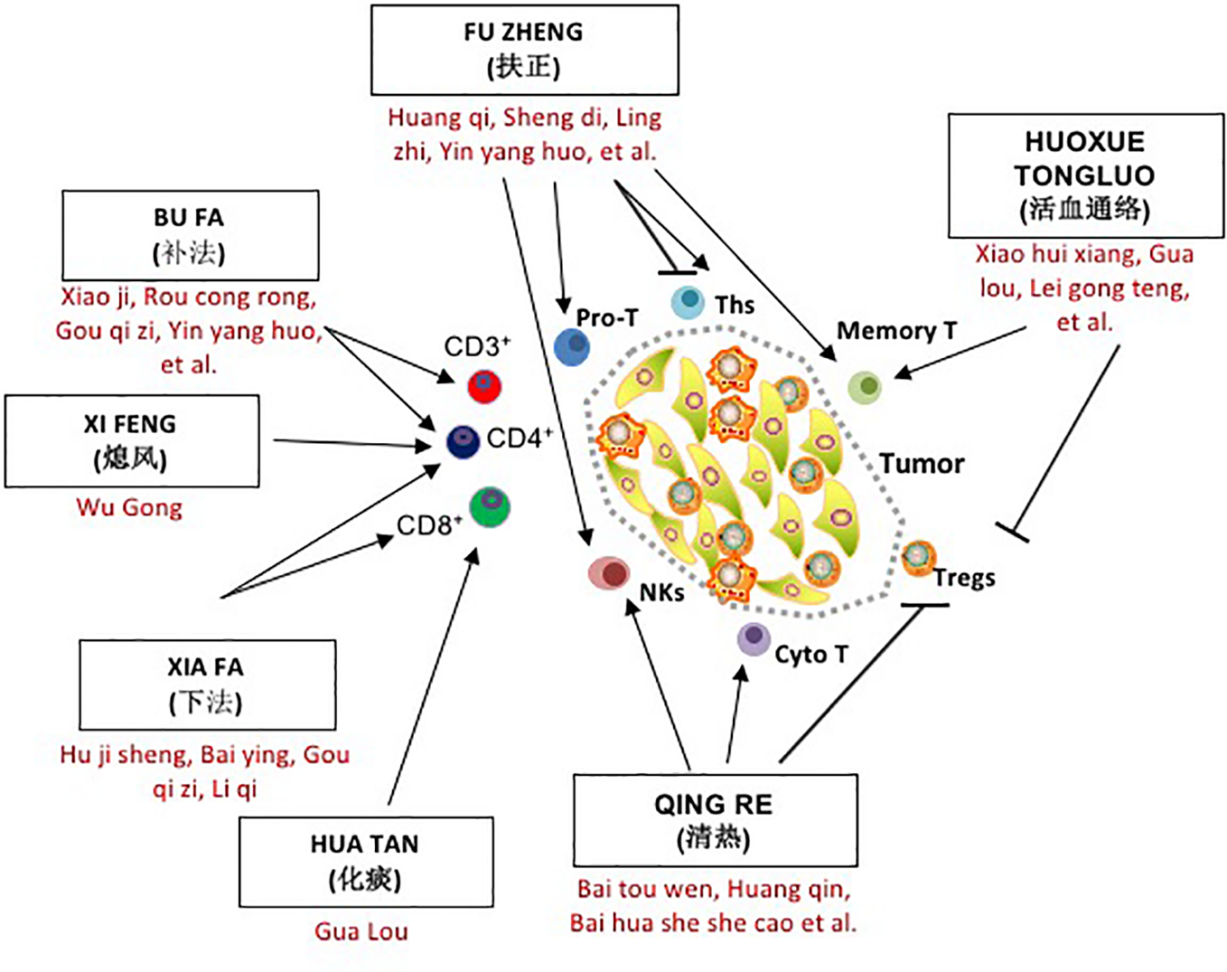
The role of seven traditional Chinese medicine (TCM) herbal methods in T cell regulation and tumor progression. Cyto T, cytotoxic T cell; Memory T, Memory T cell Tm; NKs, Natural Killer cells; Pro T, T lineage progenitor; Tc or cytotoxic T lymphocyte; Ths, helper T cell; Tregs, regulatory T cell.

As this review will demonstrate, several classical treatment methods, such as QUXIE, HUOXUE TONGLUO, and QINGRE, are widely used alongside FUZHENG in TCM herbal medicine and cancer treatment. As shown in **Figure 1**, in addition to directly inhibiting tumor proliferation and metastasis, all of these methods have an impact on the immune environment of tumor patients, especially the distribution and function of T lymphocytes, and each method has its own characteristics and emphases. Simply speaking, they are aimed at different subgroups of T cells with different mechanisms of action. Herbal treatments, dietary therapy, acupuncture, and moxibustion have long been the standard form of treatment in Chinese medical literature, and represent a rich resource for future investigation.

## Methods

An extensive search was undertaken in PubMed/MEDLINE, ScienceDirect, Ovid, PLoS, and Google using the keyword search terms TCM, Chinese herbs, T cells, tumor regulation, and cancer along with specific T cell descriptors, and both individual Chinese herbs and formulas. We selected and reviewed all relevant studies, both *in vivo* and *in vitro*, that analyzed the relationships of various Chinese formulas, herbs, and their constituents with T cells and cancer therapy over the last 25 years (1994–2019).

## Tumor Related T Cells and Their Role in Tumor Progression

Also called thymocytes (reflecting their development in the thymus), T cells or T lymphocytes are developed from hematopoietic stem cells in the bone marrow. They help protect the body from infection and help fight cancer (cancer.gov). A type of white blood cell, they search the body for cells displaying antigens either from infectious organisms or antigens arising from tumor cell mutations known as neoantigens. When the T-cell receptor (TCR) of a cytotoxic T cell recognizes and binds to an antigen, the T cell kills the cell displaying that antigen (Sarkizova and Hacohen, 2017).

**Figure 2:**
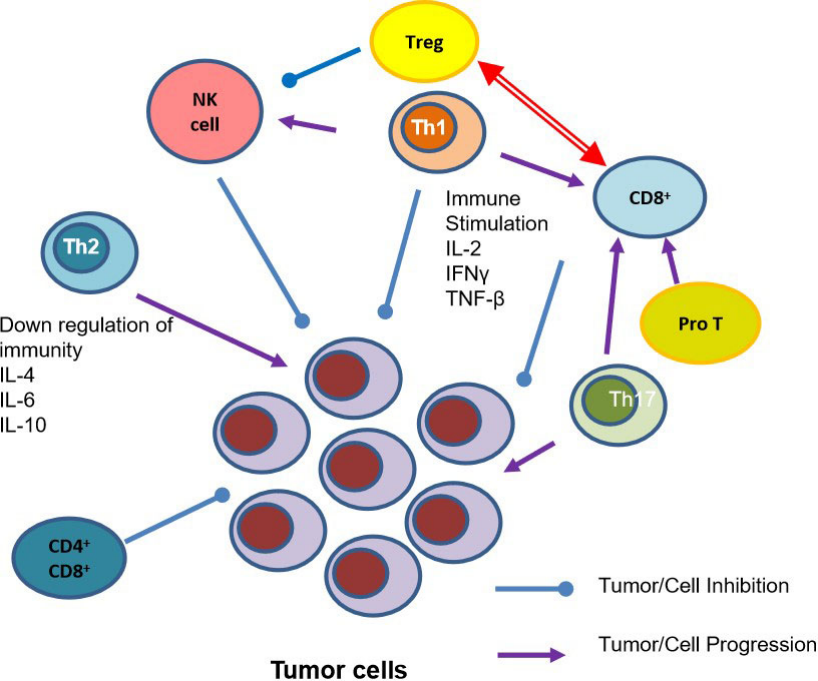
T Cell Subsets and their role in Tumor Inhibition and Progression. IFN-γ, Interferon- γ; IL-2, Interleukin-2; IL-4, Interleukin-4; IL6, Interleukin-6; IL-10, Interleukin-10; NK cell, natural killer cells; Pro T, T lineage progenitor; Th1, T helper cell 1; Th2, helper T cell 2; Th17, helper cell type 17; TNF- β, Tumor necrosis factor- β; Tregs, regulatory T cell.

Normally, the body recognizes and destroys cancer cells *via* the innate and acquired immune system, and their relative immune effector cells *via* the process of immunosurveillance (**Figure 2**). However, cancer cells may evade such immunosurveillance through immunoselection and immunosubversion. Block and Markovic (2009) suggest that multiple factors may disrupt normal immune function including production of cell surface molecules, cytokines, and growth factors by tumors in order to promote their own progression (Ha, 2009). Tumor clones, which may be more evasive to immune detection, emerge in a process called immunoediting propelled by the selective pressures of immunosurveillance (Gross et al., 2013).

As cancer develops in the human body the number of normal T cells decreases, along with B cells and natural killer (NK) cells (Noguchi et al., 2014). Further, the specific ratios between Th1 and Th2, CD4^+^ and CD8^+^, and Th17 and regulatory T Cells (Tregs) are essential in a healthy system, but as cancer develops, these ratios are dysregulated. While previous anti-genetic experience is essential in developing the body’s T cell sensitivity, additional factors such as patient and tumor genetics, and the microbiome all play essential roles as well (Lanitis et al., 2017). As the immune system continuously interacts with tumors it is essential to understand these mechanisms in developing cancer therapies.

### Pro-T Cells, Cytotoxic T Cells, and Effector T Cells

Pro-T cells or CD3^+^ cells help to activate cytotoxic T cells (CD8^+^ naive T cells) and T helper cells (CD4^+^ naive T cells). These cells are required for T cell activation, and are frequent targets of drug development. CD8^+^ T cells (cytotoxic T cells) are T lymphocytes that have the ability to recognize and kill cancer cells directly. Many studies, as outlined below, have identified Chinese herbs and formulations that promote CD8^+^ function and infiltration. Furthermore, effector T cells respond to stimulus, acting locally at sites of infection to either kill infected cells or to help other cells eliminate pathogens (Molecular Biology, 2002), and are also identified as targets for TCM herbal therapy.

### T Helper Cells

CD4^+^ T cells (T helper cells) assist white blood cells in eliminating pathogens as a part of our acquired or adaptive immune system. These cells activate cytotoxic T cells and macrophages, and aid the maturation of B cells into both plasma cells and memory cells. T helper cells suppress and regulate the immune response by secreting cytokines during the immune response and may differentiate into T_h_1, T_h_2, T_h_17, and others.

T_h_1cells are responsible for activating and regulating the development of cytotoxic T cells (CTL). They regulate the production of cytokines IFN-γ and TNF-α, and activate antigen-presenting cells (APC). The release of cytokines from T_h_1cells activates death receptors on tumor cell surfaces leading to their destruction (Knutson and Disis, 2005). T_h_1 cytokines also include IL-1, IL-2, and IL-12. Further, T_h_1 cells induce secretion of IL-1β and IL-6 in antigen-presenting macrophages, and this collaboration leads to cancer cell death (Haabeth et al., 2011).

T_h_2 cells are essential in facilitating protective type 2 immune responses (producing cytokines IL-4, IL-5, and IL-13), such as those that target parasites and facilitate tissue repair. However, they also contribute to chronic inflammatory diseases, such as asthma and allergies. Their anti-tumor effects and contributions to tumor growth remain one of the more challenging mechanisms within immunotherapy, and studies often focus on the ratio between T_h_1 and T_h_2. Factors secreted byT_h_2 such asIL-4, IL-10, and TGF-β play suppressive roles in tumor immune microenvironment, and promote tumor recurrence and metastasis (Guo et al., 2014).

T_h_17cells protect the body against pathogens as a part of the acquired or adaptive immunity. In murine studies T_h_17cells mediate the regression of tumors (Martin-Orozco et al., 2009). However, they were also shown to promote the formation of tumors when induced by colonic inflammation (Wu et al., 2009). The mechanisms by which T_h_17 cells contribute to tumor progression are not clear despite being identified in a wide variety of human tumors including ovarian, gastric, prostate, renal, and pancreatic cancers (Kim and Cantor, 2014). Studies of T_h_17 are primarily concerned with inflammatory diseases, and autoimmune disorders. Nonetheless, the results may help us understand how the regulatory effects of Chinese herbs may be useful in immunotherapy.

### CD4^+^CD8^+^ Cells and Memory T Cells

CD4^+^CD8^+^ cells are double positive T cells that express both CD4 and CD8 co-receptors. Memory T cells (TM, CD4^+^/CD44^+^) are T cells that have had interaction with specific antigens or cancer, and are able to mount a strong and rapid response to the pathogen or cancer.

### Regulatory T Cells

Regulatory T cells (Tregs) play an important role in regulating the immune system, preventing autoimmunity, and moderating inflammation. However, they also play a significant role in the development and progression of cancer *via* the suppression of tumor-specific immunity. As Tregs secrete a variety of immunosuppressive cytokines, it is essential to investigate strategies that reduce their regulatory influences while enhancing anti-tumor immunity (Ha, 2009). It has been shown that regulation of Treg cells can increase immune protection from tumor-associated antigens that are expressed as self-antigens (Kosmaczewska et al., 2008). Treg cells are characterized by CD25 and Foxp3 mRNA, a protein involved in immune responses, and the development of Treg cells.

### Natural Killer Cells

Natural Killer cells (NK) are cytotoxic lymphocytes (white blood cells) that behave like cytotoxic T cells. However, they do not require antibodies or MHC to respond to harmful cells. NK cells, also known as CD56^+^, play a major role in detecting and controlling early signs of tumors, and in killing virally infected cells (immunology.org). NK cells also produce interferon-γ, which activates M1 and T_h_1 immune responses, thus indirectly increasing cytotoxicity of cancer cells (Gross et al., 2013).

## Regulating Effects of Traditional Chinese Medicine on Tumor Related T cells

The regulatory effects of Chinese herbal medicines on the immune system of tumor-bearing organisms is commonly accepted. As shown in **Table 1**, Chinese herbs and formulas can play an important auxiliary role in the treatment of tumors by affecting many kinds of T cells, including Pro-T cells, Tregs, T helper cells, Cytotoxic T cells, and NK cells. The targeted T cells and the mechanism of change often reflect the characteristics of specific TCM treatment methods.

**Table 1:** The effects of Traditional Chinese herbal medicine on tumor related T cells.

Location	Cell phenotype	Cell name	TCM	Effects of TCM	Sources
Bone marrow	CD3^+^	Pro-T cells	Fuzheng Qingjie, Fuzheng Yiliu, Xiaoji	Increase in CD3^+^ cells	Chen et al., 2016 and Chen et al., 2017; Cao et al, 2011; Cao et al, 2012; Li L. et al., 2015
Peripheralblood circulation	CD4^+^	T Helper cell	Fuzheng Qingjie, Fuzheng Fangai,Xiaoji, Cistanche deserticola,Epimedium koreanum nakai, Glycyrrhiza uralensis, Aidi,Scolopendra subspinipes	Increase in CD4^+^ cells and CD4/CD8 ratio, produceI FN-γ and IL-2, IL-4, IL-6, IL-7	Chen et al., 2016 and Chen et al., 2017; Liu et al., 2014; Li et al., 2015; Wang et al., 2017a; Ayeka et al., 2017; Zhang et al., 2018; Guo et al., 2015; Zhao et al., 2012; Ma et al., 2014 and Ding et al., 2016
	CD4^+^/IFN-γ^+^	T_h_1	Fuzheng Yiliu*, Musca domestica*	Increase IL-2 and TNF-α	Chen et al., 2014; Sun et al., 2014 and Hou et al., 2007
	CD4^+^/IL-4^+^	T_h_2	*Aidi, Ligusticum chuanxiong, Radix astragali*,	Reduce T_h_2	Wei et al., 2003; Wang and Chen, 2009; Hou et al., 2015
	CD4^+^/IL-17^+^	T_h_17	Anemoside A3, Baicalin, Xuebijing	Reduce T_h_17	Zou et al., 2015; Ip et al., 2017; Chen et al., 2018
	CD8^+^	Cytotoxic T cell	Xiao Ai Ping, *Lycium barbarum*, Dang gui bu xue tang, *Oldenlandia diffusa*, *Carthamus tinctorius*, lectin-55, Tricosanthes kirilowi,	Increase in CD8^+^ cells, and infiltration of tumors, increase IFN-γ, IL-10	Li et al., 2013; Deng et al., 2018; Wu et al., 2009; Wang et al., 2018a; Hsieh et al., 2003; Gupta et al., 2004; Yadav and Lee, 2006; Yang et al., 2010; Bo et al., 2017; Willimott et al., 2007; Ma et al., 2008; Chang et al., 2011; Cai et al., 2011
	CD4^+^CD8^+^	Cytotoxic T cell	Shenqi Fuzheng, *Lycium barbarum, Ganoderma lucidum*, Yunzhi-Danshen	Upregulate CD3^+^, CD4^+^, CD4^+^/CD8 and NK^+^ cells	Cao et al., 1994; Bao et al., 2006; Xu et al., 2017; Saleh et al., 2017; Zhang et al., 2018; Wong et al., 2005
	CD44(hi)CD62L^+^CCR7^+^	Memory cell	*Rehmannia glutinosa*polysaccharide liposome	Induce memory cells, upregulate DC cells	Huang et al., 2016
	CD56^+^	Natural Killer cell	*Ganoderma lucidum, Radix et caulis acanthopanacis senticosi, Panacis quinquefolii radix, Scutellaria baicalensis Polygonum cuspidatum, Solanum lyratum*, Liqi	Promote NK, DC and T cells, decreaseIL-1 and TNF-α	Szolomocki et al., 2000; Gao et al., 2003; Lau et al., 2019; Ma et al., 2008; Nie et al., 2016 and Xiao et al., 2015; Chueh et al., 2015; Liu et al., 2011, Yang et al., 2010; Ji et al., 2009; Guan et al., 2013
Tumor microenvironment	CD4^+^CD25^+^Foxp3^+^	Treg	Fuzheng Fangai*, Ganoderma lucidum* polysaccharides, Fei Yanning,Artesunate	ReduceTreg, downregulation of Foxp3	Liu et al., 2014; Wang et al., 2009; Li A. et al, 2015, Guo et al. 2012); Zhang et al., 2014

### FUZHENG Combined with QUXIE

FUZHENG (扶正) therapy was developed in modern China, and utilizes herbal formulations to protect and enhance the immune system from the damage exerted by conventional therapies such as chemotherapy and radiation on patients. Though similar in concept to classical Chinese medicine tonification formulas (BUFA), it is the addition of herbs to eliminate pathogens (QUXIE) that often makes them unique in their category. The primary strategy of FUZHENG formulas combined with QUXIE (eliminating pathogens) is the positive regulation of overall immunity through the promotion of normal T lymphocyte and macrophage function, and inhibition of tumor immunosuppressive T cells such as Tregs. As FUZHENG formulas are a modern creation utilizing Chinese herbs and formulas, there is no historical record of these formulas. The formula FUZHENG QINGJIE (FZQJ) is often used along with chemotherapy in gastrointestinal cancer (Chen et al., 2017b). It was found that FZQJ could alleviate chemotherapy induced stresses including white blood cell and platelet destruction, and reduction of CD3^+^ and CD4^+^ T lymphocytes. FZQJ could also help maintain the concentration of hemoglobin, prevent the loss of body weight, and increase serum TNF-α and IL-2 levels, thus alleviating the side effects of chemotherapy (in this case cyclophosphamide or CTX). Interestingly, while FZQJ was able to inhibit the development of tumors on its own, its effects were more pronounced when used with CTX. How this occurs is still poorly understood; however, the authors suggest that the elevated levels of TNF-α (perhaps triggered by IL-2) may activate apoptosis *via* mitochondrial mediation and Bcl-2 family proteins.

Another Chinese herbal formula used in FUZHENG therapy, FUZHENG FANGAI pill (FZFA), has been shown to have few side effects with moderate but persistent efficacy. FZFA contains *Codonopsis pilosula* (tangshen, 15g), *Astragalus mongholicus* (huang qi, 30g), *lycii fructus* (gouqizi, 12g), *Polygonum multiflorum* (he shou wu, 12g), *bistort root* (quanshen, 10g), and *Chinese Actinidia root* (tenligen, 12g). Liu et al. (2014) demonstrated that FZFA along with cyclophosphamide could restore the ratio of spleen lymphocytes such as CD4^+^, and their associated cytokines IL-17, Th17, CD4^+^CD25^+^, and Foxp3^+^ Treg cells while inhibiting the inflammatory response. Remarkably, the ratio of Th17 to Treg cells approached normal ranges with this combination. Like FZQJ above, FZFA administered with CTX inhibited tumor growth and metastasis, and could improve survival rates while increasing life span, compared with the administration of CTX alone (Liu et al., 2014).

FUZHENG YILIU Decoction (*Radix ginseng* (ren shen), *Radix astragali* (huang qi), *Ganoderma lucidum* (lingzhi), *Angelica sinensis* (dang gui), and *Lycium chinense* (gou qi zi)), remarkably inhibited the proliferation of hepatoma cells, and induced apoptosis *in vivo* by promoting the production of T_h_1 cytokine IL-2 (Chen et al., 2014). A number of studies demonstrating FUZHENG Yiliu’s effects on hepatoma cells have been published showing an increase in CD3^+^, CD4^+^ and NK cells in peripheral blood with an increase in IL-2 and TNF-α, thus inhibiting hepatocellular cancer proliferation (Cao et al., 2011; Cao et al., 2012).

In a meta-analysis of 8 trials utilizing SHEN QI FU ZHENG Injection (SFI), Xu et al. (2017) found that the formula consisting of *Codonopsis* (dang shen) and *Radix astragalus* (huang qi) could improve chemotherapy outcomes, and improve immune function, upregulating CD3^+^, CD4^+^, CD4^+^/CD8^+^, and NK^+^ cells. They also found that SFI could reduce adverse side effects such as leukocytopenia, thrombocytopenia and gastrointestinal toxicity (Xu et al., 2017). Though the meta-analysis pointed out the shortcomings of the included trials (the small sample size was 772, and lacked double-blind controls), it did provide encouraging results that warrant larger, better quality clinical trials.

Li et al. (2013) demonstrated that an injection of the herbal formula XIAO AI PING (*Marsdenia tenacissima*, tong guan teng) in combination with Cisplatin promotes both the infiltration and the function of CD8^+^ T cells, thus reducing tumor growth and promoting the apoptotic effects of cisplatin in Lewis lung cancer cells. XIAO AI PING was also found to increase the infiltration of cytotoxic T lymphocytes CD8^+^ T cells (Li et al., 2013). XIAO AI PING was shown to target proliferating cell nuclear antigen (PCNA) and phosphorylated Akt, both markers for tumor growth, and increased levels C/EBP homologous protein (CHOP), a marker for tumor cell apoptosis. The study concluded that XIAO AI PING used in combination with cisplatin was more effective than either formula or chemotherapy alone. A 2019 systematic review and meta-analysis confirms this data. Wu et al. (2019) compared data from 14 studies between 2009 and 2018 utilizing a variety of chemotherapy protocols including XELOX (capecitabine and oxaliplatin), SOX (S-1 and oxaluplatin), and others in combination with XIAO AI PING. They concluded that the combination could offer an effective treatment strategy for gastric cancer patients, especially with the XIAO AI PING and XELOX pairing. They reported a reduction in leukopenia, liver and renal damage, and reduced incidence of erythrodysesthesia or hand-foot syndrome.

The formula FEI YANNING Decoction [*Radix astragali mongolici* (sheng huangqi) 40 g, *Rhizoma atractylodis macrocephalae* (baizhu) 15 g, *Succys bufo* skin (gan chenpi) 9 g, *Nidus vespae* (fengfang) 9 g, *Rhizoma paridis* (qiye yizhihua) 15 g, *Rhizoma polygonati sibirici* (huangjing) 30 g, *Herba epimedii brevicornus*(xianlingpi) 15 g, *Ganoderma lucidum* (lingzhi) 30 g] has undergone several studies related to cancer (Wang et al., 2009; Guo et al., 2012; Wang et al., 2012; etc.). Specifically, FEI YANNING (FYN) decoction was found to reduce Treg cells in the spleen and thymus, and in tumors along with significantly downregulating Foxp3 mRNA. These results suggest that FYN may be used as an adjuvant treatment to improve chemo and radiotherapy treatment, as well as anticancer vaccines.

### BUFA (Formulas and Herbs That Tonify)

BUFA formulas promote the proliferation and function of T cells and other immune positive regulatory cells through a variety of mechanisms. Important herbs that have been studied with regard to BUFA include *Radix astragalus* (huang qi), and its ability to enhance the immune system (Denzer et al., 2006); *Atractylodes rhizome* (bai zhu), which increases lymphocyte proliferation and stimulates the immune system (Son et al., 2017); and *Radix codonopsis* (dang shen), which increases red blood cells and assists in T cell formation (He et al., 2015b). Additionally, American ginseng or *Panacis quinquefolii radix* (xi yang shen) has been found to inhibit cancer of the liver (Qu et al., 2018), colon (Yang et al., 2016), and breast cancer (Yonghe, 2004). Chinese ginseng or *Radix ginseng* (ren shen) has been intensely studied as an athletic performance enhancer, and as an immunomodulator. *Radix ginseng* has also been found to have inhibitory effects on prostate cancer, hepatic carcinoma, glioblastoma, and other malignant tumors (Wang et al., 2017b). One of its constituents, ginsenoside Rh2, has been found to enhance the antitumor immunological response by increasing tumor infiltration by T lymphocytes.

The Chinese formula XIAOJI has been shown to increase both CD3^+^ and CD4^+^ cells after treatment in patients with suppressed immune systems as a result of non-small cell lung cancer. The formula contains several tonifying herbs: *Astragalus mongholicus* (huang qi), *Coriolus versicolor* (yun zhi), *Psoralea corylifolia L*. (bu gu zhi), along with herbs to clear heat and drain damp including *Hedyotis diffusa* (bai hua she she cao), *Curcuma kwangsiensis* (jiang huang), *Scorpion* (quan xie), *Centipede* (wu gong), *and Rhubarb* (da huang). Li et al. (2015) combined the formula with chemotherapy, and a transfusion of cytokine induced killer cells. Patients with this combined therapy had greater progression free survival rates, higher disease control rates, and there were no significant side effects. Li also noted that in their studies XIAOJI increased lung cancer cell apoptosis, and inhibited tumor cell growth and metastasis (Chai et al., 2014).

Zhang et al. (2018) found that *Cistanche deserticola (rou cong rong)*, when used as a replacement for alum as an adjuvant, enhanced the proliferation of T and B cells, the production of IFN-γ and IL-4 in CD4^+^ T cells, and the expression of IFN-γ in CD8^+^ T cells. Remarkably, the extraction also down-regulated Tregs, while up-regulating levels of both CD40 and CD80, signaling proteins found on the surface of antigen presenting cells, dendritic cells, B cells, and monocytes. Though this study demonstrated the use of *cistanche* as an adjuvant, it provides evidence that the polysaccharides of the herb enhance humoral and cellular immunity, and should be further investigated.

Wang et al. (2017a) found that polysaccharides from *Epimedium koreanum nakai* (yin yang huo), a Chinese tonic herb, could substantially increase macrophage activity in Lewis Lung Carcinoma-bearing mice. The results of this activity enhanced CD4^+^ differentiation, and increased immunomodulatory cytokines (IFN-γ) thus inhibiting the growth of tumors. These antitumor activities achieved host-immune regulation, and an increase in the antigen presenting function of dendritic cells.

*Lycium barbarum* (gou qi zi), an herb used to nourish the blood and mentioned earlier as a component of FUZHENG FANGAI pill, showed similar effects in an additional study (He Y. L. et al., 2005). Cao et al. (1994) found that *Lycium barbarum* polysaccharides (LBP), one of the primary constituents of gou qi zi, significantly increased the response rate and effectiveness of NK cells in advanced stage cancer patients. More recent studies demonstrated that LBP significantly increased CD4^+^ and CD8^+^ as compared with the control groups to reduce immunosuppression in H22-bearing mice, and that LBP can enhance the immune system and inhibit tumor growth (Bo et al., 2017; Deng et al., 2018; Wang et al., 2018a). In addition, goji reduces inflammation *via* regulation of the NF-κB pathway and has been shown to inhibit the growth of colon (Mao et al., 2011), and breast cancer (Li et al., 2009), along with leukemia, liver, and gastric cancer (Cheng et al., 2014).

DANG GUI BU XUE TANG (DGBXT) is a Chinese herbal formula consisting of two herbs, *Radix astragali* (huang qi) and *Angelicae sinensis radix* (dang gui), and is used to nourish blood and improve overall energy levels, especially in post-partum women. Interestingly, after just 3 weeks of oral administration, Hsieh et al. (2003) found that DGBXT “increased the population of cytotoxic T lymphocytes and NK cells,” while down-regulating T helper cells (CD4^+^/CD25^+^) in both the spleen and in tumor-draining lymph nodes. The study also demonstrated that DGBXT also increased the production of TNF-α. Chen et al. (2017) found that DGBXT could also inhibit colorectal cancer (CT-26) through autophagic processes, or cell degradation. The mechanism, LC3B lipidation, downregulation of phospho-p70^s6k^, and upregulation of Atg7, was elucidated further *in vitro*.

*Ganoderma lucidum* or Reishi mushroom (língzhī) has traditionally been used to calm the spirit in TCM, and has been known as a longevity herb for centuries. Modern research has touted numerous health benefits related to its polysaccharides and triterpenes including strengthening the immune system, and enhancing T cell and macrophage function (Wachtel-Galor et al., 2011). It has also been shown to reduce the side effects of chemo and radiotherapy (Gao et al., 2003; Kladar et al., 2016; Jin et al., 2016). In another study (Li A. et al., 2015), *Ganoderma lucidum* polysaccharides(GLPS)significantly inhibited tumor growth in hepatoma-bearing mice by increasing the ratio of T effector cells to Tregs. GLPS also increased IL-2 secretion, thus eliminating the suppression of T effector cells by Tregs. GLPS also inhibited Foxp3 mRNA expression, but in this study they further demonstrated that this occurred through an increase of miR-125b expression. Zhang et al. (2019) found similar results with *Ganoderma lucidum* polysaccharides combined with gold nanocomposites (GLP-Au). They found that the combination increased DC activation which resulted in the proliferation of CD4^+^ and CD8^+^, and when combined with the chemotherapy drug doxorubicin, GLP-Au increased CD4^+^/CD44^+^ T cells. In a clinical trial of advanced stage cancer patients, Gao et al. (2003) prescribed an extract of *Ganoderma lucidum*. After 12 weeks of oral administration they found increased levels of CD56^+^ (NK cells), plasma IL-2, IL-6, and IFN-y, along with decreased levels of IL-1 and TNF-α. Additionally, CD3^+^ (T lymphocytes), CD4^+^ (T helper cells), and CD8^+^ (T suppressor cells) showed increased levels. Chang et al. (2014) noted that *Ganoderma lucidum* stimulated the secretion of perforin and granulysin, granule proteins with lytic properties that increase NK cell cytotoxicity. However, in a Cochrane review (Jin et al., 2016), researchers noted that Zhang et al. (2000) found a negative impact on NK cell activity with *ganoderma* administration; however, this study is only available in Chinese and not available for verification.

*Glycyrrhiza uralensis*, licorice root (gan cao) is an herb used in many traditional Chinese herbal formulas to moderate and harmonize the characteristics of other herbs (Bensky et al., 2004). Ayeka et al. (2017) found that the polysaccharides of *Glycyrrhiza uralensis* significantly suppressed the growth of tumors in CT26 tumor bearing mice while activating CD4^+^ and CD8^+^. This activation of CD4^+^ and CD8^+^ increased cytokine production, specifically IL-2, IL-6, IL-7, and decreased TNF-α levels. Ayeka’s previous study (2016) also showed benefits from *Glycyrrhiza uralensis* polysaccharides on inhibiting carcinoma cell growth and upregulation of IL-7 *in vitro*. A recent study (Guo et al., 2015) showed that an extract of licorice root increased production of Foxp3^+^ regulatory T cells “after stimulation of purified naive (CD4^+^CD25^−^) T cells by CD3 and CD28 antibodies, and transforming growth factor-beta (TGFβ).” Furthermore, T cell proliferation and survival may be compromised by licorice as it reduces levels of T_h_1 cytokine IL-2. While Regulatory T cells are crucial in the prevention and regulation of inflammatory diseases, their immuno-suppressive actions are counterproductive in the treatment of cancer. Therefore, contradicting Ayeka et al.’s findings, the use of licorice extracts might be contraindicated despite its ability to enhance T helper cells.

Siberian ginseng, *radix et caulis acanthopanacis senticosi* (ci wu jia), is a qi tonic in Chinese medicine that is primarily used to strengthen the body, invigorate the blood, and improve concentration or even alleviate mild depression. Studies by Szolomocki et al. (2000) found that the herb positively affected physical fitness and lipid metabolism while stimulating T lymphocytes and natural killer cell production. Lau (2019) demonstrated that the primary active constituents from *A. senticosi* include the polysaccharides and glycopolysaccharides which stimulate or enhance T cells, cytotoxic cells, and NK cells. Lau further suggests that it is the whole-herb aqueous extract of *A. senticosi*, not extracted constituents, that may yield the most promising mechanisms for investigation.

Treatment with ACML-55 (Ma et al., 2008), a standardized extract of American ginseng or *Panacis quinquefolii radix* (xi yang shen), showed increased CD8^+^ T cells, and increased activation of innate lymphocytes, including NK cells and gamma-delta *T cells* (γδ). The study showed that the immune response was able to significantly delay tumor development in colon cancer-bearing murine models, and indicates that ACML-55 increases the antitumor response of both our adaptive immune system, and our innate immune system.

Wang and Chen (2009) observed that patients with esophageal squamous cell carcinoma (ESCC) receiving radiation therapy had significantly lower expressions of T_h_1 type transcription factor T-bet and cytokines IFN-γ and IL-2, while expressions of T_h_2 type transcription factor GATA-3 and cytokines IL-4 and IL-10 were significantly higher. However, using Aidi injection (*Mylabris* (ban mao), *Radix ginseng* (ren shen), *Radix astragali* (huang qi), and *Radix et caulis acanthopanacis senticosi* (ci wu jia)) during radiotherapy the T_h_2 factors were inhibited, suggesting that this herbal extract may reverse the T_h_2 predominant status (Wang and Chen, 2009). A retrospective study by Gang et al. (2018) further suggested that Aidi injection could be used along with chemotherapy to significantly improve Quality of Life (QoL) scores for a variety of cancers, including gastric, lung, breast, colorectal, cardiac and esophageal cancers, liver and ovarian cancer. This extensive study included 3,200 patients and further supported findings that Aidi injection could increase CD3^+^, CD4^+^, CD8^+^, NK cells, and the ratio of CD4^+^/CD8^+^, and T_h_1/T_h_2, thus enhancing cellular immunity.

### QINGRE (Formulas and Herbs That Clear Heat)

QINGRE (清热–Formulas that Clear Heat), one of the eight classical treatment methods (BA FA, 八法) in TCM, seeks to clear heat in the body in order to eliminate pathogens. From a biomedicine perspective, these herbs and formulas are considered antipyretic and anti-inflammatory, and may have antibacterial, antiviral, and antifungal effects (Chen and Chen, 2004, p. 105). A recent study (Huang et al., 2016) investigated the use of *Rehmannia glutinosa* polysaccharide liposome (RGPL), a standardized extract of the unprepared form of *rehmannia* (sheng di huang). *Rehmannia glutinosa* is traditionally used in TCM to clear heat from the body, and nourish fluids in cases of high or continuous low-grade fever, thirst, dry mouth, and constipation. In TCM, these symptoms would most commonly be associated with warm pathogenic diseases, diabetes, and in a modern context, the side effects of chemo and radiotherapy. The study found that RGPL significantly increases the percentages of central and memory cells, and effector memory cells in murine models. Of particular interest in the study was the encapsulation of RPG within a liposome in order to reduce the rate of metabolism, and therefore extend its duration of action within the body. The study demonstrated that the RGPL not only increased antigen-specific immune responses, but also triggered dendritic cell (DC) activation and immune memory. DC’s are antigen presenting cells (APC) with high levels of cell surface receptors whose main function is to capture and process antigens (Melief, 2003). The study demonstrated the upregulation of DCs, and allogenic T cell proliferation while strengthening antigen presenting functions. Though the study was primarily looking at RGPL as a vaccine adjuvant, the results provide a mechanism for increased antitumor and antiviral immune responses, and warrant further study of this important herbal extract.

A study conducted by Ip et al. (2017) found that anemoside A3 (AA3), a triterpenoid constituent of *Pulsatilla chinensis* (bai tou weng), inhibits T_h_17 cell differentiation, and reduced both T_h_1 and T_h_17 inflammatory responses. While this study measured the immunomodulatory and neuroprotective effects of AA3 in MS, its findings elucidate the effects of AA3 on the immune system, and the mechanisms by which it may help restore homeostasis *via* the downregulation of these inflammatory cytokines (T_h_1 and T_h_17).

*Scutellaria baicalensis* (huang qin) has been studied extensively, and a variety of mechanisms have been discovered. These include inhibition of PGE2 synthesis and G_0_, G_1_, G_2_, and S phase arrest. *Scutellaria* also has neuroprotective and hepatoprotective affects (Wang et al., 2018b). In TCM, it is said to clear heat from the body and is typically used to eliminate toxins. *Wogonin*, a flavonoid-like chemical compound found in *Scutellaria*, was found to induce apoptosis of tumor cells *via* DC’s, and to promote NK, DC and T cells in tumor tissue (Nie et al., 2016, and Xiao et al., 2015). Baicalin, a flavonoid found in *Scutellaria baicalensis*, significantly decreases inflammatory mediators such as TNF-α, IL-1B, and in T_h_1 related cytokines such as IL-12 and IFN-γ in rats with TNBS-induced ulcerative colitis (Zou et al., 2015). Baicalin also reduces the number of T_h_17 cells and their associated cytokines, IL-17 and Il-6. Zou states that the effects of baicalin are associated with the regulation of T_h_17 and Tregs. It is this ratio of T_h_17 to Treg that may be the mechanism with which baicalin reduces inflammation, and could lead to further research in the regulation of the tumor microenvironment.

In an older study, Yoshida et al. (1997) found that extracts of *Oldenlandia diffusa* (bai hua she she cao) enhanced the induction of alloantigen specific cytotoxic T lymphocytes, and stimulated murine spleen cells. *Oldenlandia diffusa* is used in TCM to clear heat and eliminate toxins, and as such is often used in herbal formulas to treat cancer in China. Studies consistently show its pro-apoptotic effects *via* caspase-dependent apoptosis and other mechanisms (Gupta et al., 2004; Yadav and Lee, 2006; Willimott et al., 2007; Yang et al., 2010).

*Musca domestica* (MDPF, also known as wugu chong), the larvae of domestic houseflies, has been used in China for cancer treatment along with osteomyelitis, decubital necrosis, and other diseases. Sun et al. (2014) found that oral administration of MDPF for 10 days could not only inhibit the growth of S180 sarcoma, but also enhance splenocyte proliferation, and both NK cell and CTL activity from the splenocytes. Furthermore, MDPF significantly promoted T_h_1 transcription factors T-bet and STAT-4 in splenocytes, while enhancing Ig2, IgG2a, and IgG2b antibody levels. Sun et al. speculated that MDPF may trigger T_h_1 specific cell-mediated immunity. Previous studies showed that MDPF inhibited bacterial pathogens such as *e.coli*, and inhibited tumor growth in human colon cancer CT26 cell lines (Hou et al., 2007).

Artesunate (ART), is an artemisinin based compound derived from *Artemisia annua* (huang hua hao), an herb that is traditionally used to treat malaria as an anti-inflammatory and antifebrile medicinal. A 2014 study showed that ART was capable of inhibiting orthotopic tumor growth in human cervical cancer cells while decreasing Foxp3 expression in T cells, and facilitated the reduction of Tregs (Zhang et al., 2014). The study demonstrates that ART may be effective for use in the treatment of cervical cancer utilizing immunotherapy. Another study utilized ART as an adjuvant treatment in esophageal cancer with radiotherapy (Fei et al., 2018). It was found that administration of ART inhibited esophageal cancer cell growth (TE-1 cell line), and increased the sensitivity to radiotherapy. Interestingly, pretreatment with ART enhanced the apoptotic effects of radiotherapy, and downregulated or reversed radiation induced G_2_/M arrest, the process by which damaged cells are not able to initiate mitosis.

### HUOXUE TONGLUO (Formulas and Herbs That Promote Blood Circulation and Remove Obstructions)

Herbs and formulas to promote blood circulation and remove obstructions from the collateral channels (HUOXUE TONGLUO, 活血通络) may also be included in TCM cancer treatment. HUOXUE TONGLUO herbs may dilate blood vessels, and have anticoagulant and antiplatelet effects. They may also provide analgesic and anti-inflammatory effects (Chen and Chen, 2004, p. 612). *Ligusticum chuanxiong* is one of the primary herbs used in TCM to improve blood circulation. Wei et al. (2003) found that a preparation of the herbs *Radix astragali* (huang qi), and one of the primary constituents of *Ligusticum chuanxiong* (chuan xiong), tetramethylpyrazine (TTMP), could enhance levels of T_h_1 cytokines (IFN-γ and IL-2), and reduce T_h_2 cytokines in cultured peripheral blood mononuclear cells (PBMC) of lung cancer patients. Additional studies showed that the polysaccharides of *Radix astragali* reduced the percentage of T_h_2 cells along with Tregs, and restored the ratio of T_h_1 and T_h_2 (Hou et al., 2015).

Chang et al. (2011) found that extracts of *Carthamus tinctorius* (hong hua), another important herb used to increase blood circulation in TCM, increased production of cytotoxic CD8^+^ T cells while also enhancing dendritic cell cancer vaccines. In the study, a *Carthamus tinctorius* (CT) treated DC vaccine also stimulated splenic T lymphocytes to secrete IFN-γ and IL-10, and heightened expressions of CD86, MHC-1, and MHC-II antigen-presenting cells. Studies have further demonstrated the antitumor effects of CT polysaccharides (Wakabayashi et al., 1997; Ando et al., 2002; Shi et al., 2010).

In another study by Chueh et al. (2015), a crude extract of *Polygonum cuspidatum* (hu zhang) was found to promote natural killer cell activity of splenocytes, and promoted B cell proliferation at specific doses (200 mg/kg), but did not promote T cell proliferation in leukemic mice. *Polygonum cuspidatum* is typically used in Chinese medicine as a laxative due to the natural presence of emodin. Recent studies have focused on its resveratrol content, though Kimura and Okuda (2001) note that resveratrol has no effect on the levels of CD4^+^, CD8^+^, or NK cells.

Further, *Tripterygium wilfordii* or Thunder God Vine (lei gong teng) has had an almost meteoric rise in research circles for its positive effects on arthritis, and in fighting cancer. The herb is used in Chinese medicine to increase blood circulation and remove obstructions (HONGXUE TONGLUO), but rarely as it has potential toxic side effects (Chen, 2004; Zhao et al., 2015). More than 100 different compounds have been isolated from *Tripterygium wilfordii* (Liu et al., 2011), and research aimed at understanding their actions is underway. One such study suggests that leukemic T-lymphocyte apoptosis is induced by triptolide, a diterpenoid epoxide, and is related to cell cycle G1 phase arrest (Zhang et al., 2015).

XUEBIJING (XBJ) injection is commonly-used to treat sepsis and septic shock. The formula contains five Chinese herbs, *Radix Angelicae Sinensis* (dang gui), *Rhizoma Chuanxiong* (chuanxiong*), Radix Paeoniae Rubra* (chi shao), *Radix Salviae Miltiorrhizae* (dan shen), and *Flos Carthami* (hong hua). From a TCM perspective, XUEBIJING was derived from Xuefu zhuyu decoction, and invigorates blood circulation, dispels stasis, cools and detoxifies blood. In a meta-analysis and systematic review, Chen et al. (2018) found that XBJ could inhibit inflammation through the regulation of Tregs and T_h_17, and reduced inflammatory cytokines TNF-α and IL-6. In another study, Jiang et al. (2013) identified the constituents by which the inflammatory process was downregulated *via* the NF-kB pathway by senkyunolide I, safflor yellow A, oxypaeoniflorin, and benzoylpaeoniflori (Jiang et al., 2013). Though these studies demonstrate the use of XBJ to reduce sepsis and septic shock, the mechanisms by which they operate *via* T cells encourage further research and investigation.

Several studies have shown the efficacy of YUNZHI-DANSHEN decoction (Wong et al., 2005; Bao et al., 2006; Saleh et al., 2017). This simple formula consists of *Coriolus versicolor* (yun zhi, commonly called Turkey Tail mushroom), and *Salvia miltiorrhiza* (dan shen). Yun zhi is traditionally used in TCM to promote the immune system, while dan shen is typically used to improve circulation. Wong et al. (2005) found in a clinical trial of post-treatment breast cancer patients that yunzhi-danshen decoction could significantly elevate CD4^+^ T cells along with B-lymphocytes after just 6 months of oral administration. The formula also improved the ratio of CD4^+^/CD8^+^. Further, plasma sIL-2R (a membrane receptor) concentration was significantly reduced, showing that consumption of yunzhi-danshen decoction orally can promote immune function and response in post-treatment breast cancer patients. Interestingly, YUNZHI-DANSHEN decoction was also effective in reducing lymphopenia during radiotherapy of nasopharyngeal carcinoma patients during a 16-week trial (Bao et al., 2006).

### XIAFA (Formulas and Herbs That Drain Downward)

XIAFA, another classical treatment method in TCM eight methods, cleanses the intestines and stomach, so that the pathogenic factors can be removed from the body, such as food, dry excrement, excess heat, cold accumulation, blood stasis, phlegm knot, and effusion. Lectin-55 is an active constituent of Chinese mistletoe (hu ji sheng or *herb visci*). It is primarily used in Chinese medicine to dispel or drain damp, terminology often used to describe symptoms of rheumatoid arthritis, and soreness/weakness of the tendons in the low back or knees. Ma et al. (2008) demonstrated *herb visci’s* ability to enhance CD4^+^ and CD8^+^ T cell proliferation in colon cancer, and were able to demonstrate increased production of both antigen-specific CD8^+^ T cells, and cytokine IFN-γ. Lectin’s represent an important focus in cancer research as they are present in many plant species, and may increase both apoptosis and autophagy (Yau et al., 2015).

Natural Killer cells can be promoted with an extract of *Solanum lyratum* (bai ying). A traditional herb used to clear heat and dampness from the body, it is now used to regulate the immune system and treat allergies. Liu et al. (2011) found that the main active fraction, n-butanol extract (BESL), promoted NK cell, and CTL activity while significantly inhibiting the growth of S180 sarcoma transplanted mice. BESL also promotes splenocyte proliferation, and in turn increases IL-2 and IL-y production improving the immune response. Guan et al. (2013) confirmed *Solanum lyratum’s* immunomodulatory effects, and its ability to inhibit the growth of tumors in S180 sarcoma transplanted mice. Additional studies by Yang et al. (2010) showed that *Solanum lyratum* could increase both macrophage and natural killer cell activity in murine models bearing WEHI-3 murine leukemia cells, and may act as an anticancer agent in patients (Yang et al., 2012).

Further studies suggest that Liqi, another traditional herbal formula used for centuries in China to treat cancer, increases the activity of both NK cells and TNF-α, while increasing IL-2 activity and production (Ji et al., 2009). Liqi was also effective in regulating T lymphocyte subpopulations, and inhibited Lewis lung carcinoma metastasis. The formula consists of *Poncirus trifoliate (L.) Raf* (zhi ke)*, Akebia Trifoliate Koidz* (san ye mu tong), *Citrus medica* var. *sarcodactylis Swingle* (fo shou), and *Saussurea lappa* (yun mu xiang), and contains a wide variety of active constituents including coumarins, flavonoids, terpenoids, triterpenes and triterpenoids, polysaccharides, and sesquiterpenes.

### HUATAN (Formulas and Herbs That Transform Phlegm)

Phlegm, an accumulation of damp, is often a part of the TCM diagnosis related to cancer as phlegm is considered a primary cause of masses in the body. Cai et al. (2011) found that *Trichosanthes kirilowi* (gua lou), an herb used to transform and remove damp phlegm in TCM, increased the percentage of effector T cells, including CD4^+^ and CD8^+^ T cells which produce cytokine interferon-gamma (IFN-γ). The increased immune response to tumor growth occurs *via* the interaction between tumor suppression in lung cancer 1 (TSLC1) and class 1-restricted T cell-associated molecule (CRTAM), and results in inhibited cell proliferation and increased apoptosis. Interestingly, *Trichosanthes kirilowi* has been shown to augment T_h_2 cells, and to induce HLA-associated immune suppression in bone marrow derived cells, and to decrease CD3^+^CD8^+^ T cells in the lymph nodes and spleen of naive mice although Cai et al. demonstrated this was not the case in tumor-bearing mice.

### XI FENG (Formulas and Herbs That Extinguish Wind)

XI FENG means extinguish wind, this method normally uses animal derived proteins or alkaloids to remove pathogenic factors. Zhao et al. (2012) found that polysaccharides from centipede, *Scolopendra subspinipes* (wu gong), could also increase the percentage of CD4^+^, B cells, and NK Cells, while regulating the ratio of T_h_1 and T_h_2 cytokines in sarcoma S180 bearing mice, thus inhibiting the growth of S180. *Scolopendra subspinipes* is typically used in TCM to stop tremors and spasms, but is also used to attack toxins in the body and dissipate nodules. The same study revealed that *Scolopendra subspinipes* polysaccharides also prolonged the survival time of hepatoma H22 bearing mice. Ma et al. (2014) further elucidated the effects of *Scolopendra subspinipes* on cancer cells, specifically A375 human melanoma cells, finding that the extract could inhibit cell growth at the DNA synthesis phase (S-phase), and induce cell apoptosis. And Ding et al. (2016) repeat this finding of cell cycle arrest and apoptosis in their investigation of human glioma (U87) cancer cells with isoquinoline alkaloids 1-2 isolated from *Scolopendra subspinipes*.

## Conclusion and Perspectives

The vast majority of traditional Chinese medicine formulas are extracted from one or more medicinal herbs with multiple bioactive constituents. Understanding these constituents, their interactions, and biological pathways is key to not only developing targeted cancer therapies but also to create a bridge between allopathic and TCM practitioners.

FU ZHENG therapy and other herbal treatment methods can be beneficial when used in conjunction with conventional cancer treatments (Nie et al., 2016; Xiang et al., 2019). However, TCM herbal therapy, personalized cancer therapy, and immunotherapy strive to enhance a cancer patient’s own ability to fight cancer. As these therapies gain a stronger foothold in cancer treatment strategies (especially T cell-based therapy), Chinese medicine practitioners are presented with a unique opportunity to improve TCM’s mainstream role in clinical cancer management. Moreover, TCM differential diagnosis may be used as a significant tool to create individualized treatment plans and protocols, thus guiding personalized cancer therapy. Utilizing these unique diagnostic tools and the Eight Treatment Methods as presented above may further enhance clinical outcomes.

As researchers discover the mechanisms and effects of Chinese medicinal herbs on specific body systems such as T cells (summarized in **Table 1**), we can form consensus treatments and offer more valuable insights into cancer patient care. Looking forward, future investigations should be directed at TCM’s effects on cancer stem cells and the tumor microenvironment (Qi et al., 2015; Xiang et al., 2019), as these represent exciting potential for the discovery of new multi-targeted cancer drugs and therapies.

## Author Contributions

J-LG conceived and designed the project. RH wrote the manuscript. C-YL, KH, XW, B-CH, and T-CH edited and commented on the manuscript.

## Funding

This work was supported by the National Natural Science Foundation of China (81473575 to J-LG) and the Zhejiang Provincial Natural Science Foundation of China under Grant No. LY19H280010 to KH. T-CH was supported by the Mabel Green Myers Research Endowment Fund and The University of Chicago Orthopaedic Surgery Alumni Fund. Funding sources were not involved in the study design; in the collection, analysis and interpretation of data; in the writing of the report; and in the decision to submit the paper for publication.

## Conflict of Interest

The authors declare that the research was conducted in the absence of any commercial or financial relationships that could be construed as a potential conflict of interest.
